# Cognition and Gait Show a Selective Pattern of Association Dominated by Phenotype in Incident Parkinson’s Disease

**DOI:** 10.3389/fnagi.2014.00249

**Published:** 2014-10-21

**Authors:** Sue Lord, Brook Galna, Shirley Coleman, Alison Yarnall, David Burn, Lynn Rochester

**Affiliations:** ^1^Institute of Neuroscience, Newcastle University, Newcastle upon Tyne, UK; ^2^UK and Industrial Statistics Research Unit, Newcastle University, Newcastle upon Tyne, UK

**Keywords:** Parkinson’s disease, gait, cognition

## Abstract

Reports outlining the association between gait and cognition in Parkinson’s disease (PD) are limited because of methodological issues and a bias toward studying advanced disease. This study examines the association between gait and cognition in 121 early PD who were characterized according to motor phenotype, and 184 healthy older adults. Quantitative gait was captured using a 7 m GAITrite walkway while walking for 2 min under single-task conditions and described by five domains (pace, rhythm, variability, asymmetry, and postural control). Cognitive outcomes were summarized by six domains (attention, working memory, visual memory, executive function, visuospatial function, and global cognition). Partial correlations and multivariate linear regression were used to determine independent associations for all participants and for PD tremor-dominant (TD) and postural instability and gait disorder (PIGD) phenotypes, controlling for age, sex, and premorbid intelligence using the national adult reading test. Cognitive and gait outcomes were significantly worse for PD. Gait, but not cognitive outcomes, was selectively worse for the PIGD phenotype compared with TD. Significant associations emerged for two gait domains for controls (pace and postural control) and four gait domains for PD (pace, rhythm, variability, and postural control). The strongest correlation was for pace and attention for PD and controls. Associations were not significant for participants with the TD phenotype. In early PD, the cognitive correlates of gait are predominantly with fronto-executive functions, and are characterized by the PIGD PD phenotype. These associations provide a basis for understanding the complex role of cognition in parkinsonian gait.

## Introduction

Gait disturbance is a cardinal feature of Parkinson’s disease (PD) and manifests as one of the earliest symptoms. Although the initial effect of dopaminergic medication on gait is dramatic (Morris et al., 1994), it attenuates over the course of the disease with the most debilitating features of gait dysfunction such as festination, freezing of gait, and falls staying refractory to medication. Disease progression is considered to be slower for people with PD who present with tremor-dominant (TD) phenotype compared with the postural instability and gait disorder (PIGD) phenotype where greater decline is evident not just for gait but for cognitive and other non-motor features (Alves et al., 2006; Burn et al., 2006).

Gait, which is considered a mostly automatic motor skill, is impaired in PD and cognitive control and is required to compensate for gait deficit (Yogev et al., 2005). This becomes difficult to maintain as disease advances and gait impairment reaches a threshold beyond which motor (or cognitive) compensation is no longer effective. Because of this, the relationship between gait and cognition in PD is clearly evident. The cognitive correlates of gait are dominated by attention and executive function, which chiefly correspond with gait velocity and stride length (Rochester et al., 2004; Lord et al., 2010) although other associations have been reported (Lord et al., 2011). Correlates are generally stronger in PD compared to controls, and further strengthened for PD with mild cognitive impairment (MCI) (Amboni et al., 2012) and also in more advanced disease (Bohnen et al., 2013). Mechanistic and imaging studies support a role for cognition in PD gait (Maillet et al., 2012), although as with behavioral research it has mostly been conducted in advanced disease cohorts. In early PD, cognitive impairment in attention, executive function, and working memory is mediated via dopaminergic frontostriatal networks, and further posterior cortical dysfunction, perhaps determined by cholinergic deficits, are expressed as amnestic dysfunction (Yarnall et al., 2011; Alves et al., 2013).

Recent evidence suggests that selective features of gait are underpinned by neurochemical deficits also involved in determining cognitive function, such as cortical cholinergic function (Rochester et al., 2012; Amboni et al., 2013; Bohnen et al., 2013). Gait metrics may therefore act as sensitive biomarkers and have utility in identifying and predicting cognitive risk factors in PD as well as in healthy aging and dementias (Verghese et al., 2007; Amboni et al., 2012; Hausdorff and Buchman, 2013). However, few studies have taken a systematic approach using a comprehensive range of gait and cognitive characteristics to explore the independent and selective associations between these complex functions. Gait and cognition are non-unitary constructs, and measurement tools need to reflect this diversity. The aim of this study was to explore the associations between gait and cognition in early PD using a comprehensive quantitative evaluation of gait. We hypothesized that independent gait and cognitive characteristics would show a specific rather than global pattern of association; this would differ in PD and controls highlighting the predominant frontostriatal cognitive disturbance present in early PD; and the association would become stronger in PIGD cohort because of shared pathological risk factors for faster rate of motor and cognitive decline.

## Method

### Participants

People with PD were recruited within 4 months of diagnosis of idiopathic PD as part of an incident cohort study [incidence of cognitive impairment in cohorts with longitudinal evaluation – Parkinson’s disease (ICICLE-PD)] conducted between June 2009 and December 2011(Khoo et al., 2013). PD was diagnosed by a movement disorders specialist according to the UK Parkinson’s Disease Brain Bank criteria (Gibb and Lees, 1988) and were excluded if they presented with memory impairment [mini mental state exam (MMSE) ≤24] (Folstein et al., 1975), dementia with Lewy bodies, drug induced parkinsonism, “vascular” parkinsonism, progressive supranuclear palsy, multiple system atrophy, cortico-basal degeneration, or poor command of English.

To provide a comparison with normal aging and to generate normative values for cognitive tests, controls of similar age and sex to patients were recruited from community sources. Inclusion criteria were (1) >60 years of age; (2) able to walk independently without a walking aid; and (3) no significant cognitive impairment, mood, or movement disorder. The study was approved by the Newcastle and North Tyneside Research Ethics Committee and all participants gave informed consent. Clinical testing took place 1 h after medication intake to ensure optimal function. Full details of the recruitment process are described elsewhere (Khoo et al., 2013).

#### Clinical assessment

Age, sex, and national adult reading test (NART) (Nelson, 1982) were reported. Disease severity was measured using the movement disorder society (MDS)-revised united Parkinson’s disease rating scale (UPDRS) (Goetz et al., 2008) and TD and PIGD phenotypes derived from UPDRS scores (Stebbins et al., 2013). For the timed chair stand (a proxy for bradykinesia and muscle strength), participants were asked to stand up from a seated position with arms folded across their chest and sit down five times, as quickly as possible (Podsiadlo and Richardson, 1991). Balance self-efficacy was measured using the activities balance self confidence scale (Powell and Myers, 1995), depression with the geriatric depression scale (Schrag et al., 2007), and physical fatigue was measured with the multidimensional fatigue inventory (Smets et al., 1995). Levodopa equivalent daily dose (LEDD) scores were calculated for each patient (Tomlinson et al., 2010).

#### Gait assessment and outcomes

Gait was assessed using a 7 m long × 0.6 m wide instrumented walkway (Platinum model Gaitrite, software version 4.5, CIR systems, USA) under single-task conditions. Participants were instructed to walk at their comfortable walking pace for 2 min around a 25 m oval circuit (Galna et al., 2013). To aid interpretation of gait outcomes, we used a model of gait that we developed for older adults and subsequently validated in PD. The model comprises 16 gait variables describing 5 independent domains of gait: *pace* (step velocity, mean step length, swing time variability), *rhythm* (step time, swing time, stance time), *variability* (step velocity variability, step length variability, step time variability, stance time variability), *asymmetry* (swing time asymmetry, step time asymmetry, stance time asymmetry), and *postural control* (step length asymmetry, mean step width, step width variability) (Lord et al., 2013a).

#### Cognitive function

Six domains of cognition were assessed including *global cognition*, which was measured with the MoCA (Nasreddine et al., 2005). For the five remaining domains, neuropsychological tests were selected according to earlier work (Rochester et al., 2004; Lord et al., 2010) and extended to include other cognitive functions we hypothesized to relate to gait. Individual test scores for neuropsychological tests were converted to *z* scores relative to the control cohort and combined into compound scores (mean of *z* scores) for the following domains: *attention* was assessed by the mean of simple reaction time, choice reaction time, and digit vigilance time using the cognitive drug research (CDR) computerized battery (Wesnes, 2002) and also *fluctuating attention* was measured using the CV of the same variables for descriptive purposes only. We explored *fluctuating attention* in descriptive analysis as a core cognitive function, which is sensitive to age-related cognitive decline (Salthouse, 1996b) and a characteristic of dementia seen in PD (Emre, 2003). Because of the high correlation between *attention* and *fluctuating attention* we combined these two domains for subsequent analysis. *Visual memory* was measured with spatial recognition memory (SRM), pattern recognition memory (PRM), and paired associates learning (PAL) tasks from the computerized CANTAB battery (Robbins et al., 1994). *Executive function* was assessed with the modified one touch stockings (OTS) version of the Tower of London task from CANTAB (Robbins et al., 1994), and also included measures of response inhibition, learning, and task switching from Hayling and Brixton (Burgess and Shallice, 1997), phoenemic fluency (words beginning with “F” in 1 min) (Benton, 1968), and semantic fluency (animals in 90 s) (Goodglass and Kaplan, 1972). *Visuospatial function* was measured using a composite score of intersecting pentagons from the MMSE (Folstein et al., 1975), which was scored on a three point scale (Ala et al., 2001), and item 1 from the Montreal cognitive assessment (MoCA) (trail making A, cube copying, and clock drawing) (Nasreddine et al., 2005). *Working memory* was assessed using the forward digit span from the Wechsler adult intelligence scale (Wechsler, 1958).

#### Data analysis

Univariate and bivariate analyses were used to describe the data, which were inspected for normality and transformed where required. PD participants were grouped into TD and PIGD phenotypes [data from indeterminate (ID) participants (*n* = 13) were not analyzed] (Goetz et al., 2008). Student’s *t-*test and Chi-square test were used to examine difference between control and PD participants and between PIGD and TD for all outcomes, including all gait characteristics. Scores for cognitive and gait domains were calculated so that positive values indicate better function. Associations between cognitive and gait domains were first examined using partial correlations, controlling for age, sex, and NART. In preliminary analysis, we included height as a covariate but this did not influence findings and was not included in subsequent analysis.

##### Multivariate linear regression analysis

Multivariate linear regression analysis further identified independent associations between gait and cognitive domains for controls, PD participants, and TD and PIGD motor phenotype. For Model^a^, five independent models were examined, with each gait domain entered as the dependent variable (*pace*, *rhythm*, *asymmetry*, *variability*, and *postural control*) and independent variables entered in two stages. Age, sex, and NART scores were entered in the first block and all cognitive domains other than *global cognition* were entered as the second group using a stepwise procedure. For Model^b^, five gait domains were again entered as dependent variables; however, for this analysis only *global cognition* (MoCA) was entered as the cognitive variable. MoCA was of interest because it defines MCI (Level I) in PD (Litvan et al., 2012), and because global cognition has been shown to have a selective effect on gait in older adults (Watson et al., 2010). Univariate, bivariate, and regression data were examined for distribution, skewness, and residuals to ensure assumptions of normality were not violated. Due to the exploratory nature of study and limited sample size for phenotype analysis the alpha level was set at 0.05. SPSS v 21 was used to analyze the data.

## Results

Incidence of cognitive impairment in cohorts with longitudinal evaluation – Parkinson’s disease identified 165 incident PD cases of which 150 were referred to ICICLE-GAIT. Consent was obtained for 127 participants and 121 were accepted for baseline assessment. The average age of the 29 ICICLE-PD participants (18 men and 11 women) who did not take part was 67.8 and 71.1 years, respectively. Control participants (*n* = 184) were significantly older and there were more females. PD participants in this newly diagnosed cohort presented with mild to moderate PD, with most classified as H&Y I and II (*n* = 100; 82.6%). With respect to motor phenotype, 55 (45.4%) were classified as TD and 53 (43.9%) as PIGD. Compared to controls, PD participants were more fatigued and depressed and had lower scores for balance self-efficacy. NART scores were not significantly different (Table 1).

**Table 1:** **Clinical characteristics for all participants**.

Characteristic	Controls (*n* = 184) Mean (SD)	PD (*n* = 121) Mean (SD)	P*	TD (*n* = 53) Mean (SD)	PIGD (*n* = 55) Mean (SD)	*P*
Male/female (*n*)	78/106	81/40	<0.001	31/22	40/18	0.156
Age (years)	69.4 (7.7)	67.0 (10.4)	0.024	66.1 (12.1)	67.3 (8.8)	0.575
Height (m)	1.67 (0.09)	1.69 (0.08)	0.046	1.69 (0.08)	1.70 (0.08)	0.637
NART	116.9 (7.6)	114.9 (11.0)	0.071	115.0 (11.0)	114.5(11.7)	0.825
LEDD (mg day^−1^)	–	124.6 (146.0)	–	145.9 (155.5)	212.4 (131.0)	0.018
UPDRS III	–	25.5 (10.4)	–	25.9 (10.3)	23.6 (10.3)	0.244
Hoehn and Yahr stage		I (28); II (72); III (21)	–	I (18); II (34); III (1)	I (9); II (29); III (17)	<0.0001
Sit to stand	12.4 (3.9)	13.7 (4.2)	0.173	13.3 (4.7)	13.6 (3.9)	0.750
ABCs (0–100)	91.8 (10.9)	82.5 (18.9)	<0.001	85.9 (17.6)	81.0 (18.6)	0.162
MFI–total fatigue (0–100)	38.1 (13.8)	49.9 (17.4)	<0.001	44.9 (15.8)	52.4 (17.2)	0.022
GDS (0–15)	1.1 (1.8)	2.6 (2.1)	<0.001	2.2 (2.3)	2.8 (2.0)	0.176

*NART, national adults reading test; LEDD, levodopa daily dose equivalent; UPDRS III, united Parkinson’s disease rating scale; ABCs, activities balance confidence-specific scale; MFI, multidimensional fatigue inventory; GDS, geriatric depression scale*.

*Student’s *t*-test apart from sex (Chi-square).

### Gait outcomes: Effect of pathology and motor phenotype

All gait variables apart from step velocity variability, swing time, and step width were significantly different between controls and PD participants. PD participants walked more slowly, with increased asymmetry, reduced step length, and overall a more variable gait. Gait outcomes were also worse for PIGD participants who walked with a significantly slower, more variable gait, and with a shorter step length compared to TD. For PIGD, 5 of the 16 gait characteristics were significantly worse: gait speed, step length, step time variability, swing time variability, and stance time variability (Table 2).

**Table 2:** **Gait characteristics for all participants and for PIGD and TD motor phenotype**.

Gait domain and characteristics	Controls (*n* = 184) Mean (SD)	PD (*n* = 121) Mean (SD)	*P*	TD (*n* = 53) Mean (SD)	PIGD (*n* = 55) Mean (SD)	*P*
Pace
Step velocity (m⋅s^−1^)	1.26 (0.19)	1.12 (0.21)	<0.001	1.19 (0.20)	1.08 (0.20)	0.005
Step length (m)	0.67 (0.08)	0.62 (0.10)	<0.001	0.65 (0.09)	0.59 (0.09)	0.002
Swing time variability (ms)	15.1 (5.4)	17.6 (6.0)	<0.001	15.8 (5.4)	18.7 (6.3)	0.007
Rhythm
Mean step time (ms)	536.9 (46.9)	560.2 (48.4)	<0.001	555.2 (45.1)	559.3 (50.9)	0.662
Mean swing time (ms)	386.7 (30.1)	391.5 (32.9)	0.190	393.5 (30.5)	387.9 (34.0)	0.373
Mean stance time (ms)	687.6 (71.6)	729.4 (76.6)	<0.001	717.7 (71.8)	730.9 (80.3)	0.373
Variability
Step velocity variability (m⋅s^−1^)	0.052 (0.012)	0.054 (0.017)	0.413	0.052 (0.013)	0.055 (0.002)	0.421
Step length variability (m)	0.019 (0.005)	0.022 (0.008)	<0.001	0.021 (0.007)	0.023 (0.007)	0.187
Step time variability (ms)	16.3 (5.7)	19.0 (6.6)	<0.001	17.3 (6.2)	19.8 (6.6)	0.032
Stance time variability (ms)	19.6 (8.1)	23.4 (10.09)	<0.001	21.1 (9.2)	24.7 (10.2)	0.038
Asymmetry
Swing time asymmetry (ms)	8.9 (9.4)	17.2 (20.1)	<0.001	14.0 (13.3)	21.6 (25.7)	0.071
Step time asymmetry (ms)	11.2 (10.7)	22.6(27.4)	<0.001	18.8 (17.5)	26.6 (34.8)	0.371
Stance time asymmetry (ms)	8.8 (9.3)	16.8 (19.7)	<0.001	14.3 (12.6)	20.7 (25.3)	0.220
Postural control
Step length asymmetry (m)	0.020 (0.016)	0.026 (022)	0.014	0.022 (0.020)	0.028 (0.024)	0.153
Mean step width (m)	0.089 (0.024)	0.094 (0.030)	0.365	0.088 (0.027)	0.096 (0.032)	0.159
Step width variability (m)	0.022 (0.005)	0.018 (0.005)	<0.001	0.018 (0.009)	0.019 (0.006)	0.294

### Cognitive outcomes: Effect of pathology and motor phenotype

Scores for global cognition (MoCA) were significantly worse for PD (*P* < 0.001) but no different between phenotypes. Cognitive outcomes were significantly worse for PD compared to controls apart from choice reaction time and reaction time variability. Between-group differences for reaction time and Brixton score were significant but not as strong as other significant findings (*P* = 0.029 and 0.020, respectively). Intersecting pentagons reached a ceiling effect for both groups with only 16 (8.7%) control participants and 12 (9.9%) PD participants scoring fewer than the maximum of two points. Cognitive scores did not differ significantly for motor phenotype apart from semantic fluency (*P* = 0.046) with lower scores for the PIGD phenotype (Table 3).

**Table 3:** **Neuropsychological characteristics for all participants and for PIGD and TD motor phenotype**.

Neuropsychological test	Controls (*n* = 184) Mean (SD)	PD (*n* = 121) Mean (SD)	*P*	TD (*n* = 53) Mean (SD)	PIGD (*n* = 55) Mean (SD)	*P*
Global cognition
MoCA (*n* = 89 controls, *n* = 115 PD)	27.1 (2.4)	25.1 (3.5)	<0.001	25.2 (3.7)	25.2 (3.2)	0.999
Working memory
Forward digit span	6.1 (1.2)	5.7 (1.1)	0.004	5.9 (1.1)	5.6 (1.1)	0.136
Power of attention	1303.3 (155.9)	1357.0 (200.6)	0.010	1341.3 (163.3)	1352.6 (228.1)	0.984
Reaction time (mean)	326.3 (63.7)	347.1 (101.1)	0.029	336.8 (57.4)	352.1 (135.1)	0.458
Choice reaction time (mean)	521.7 (73.9)	528.9 (84.0)	0.436	527.2 (83.4)	479.4 (56.3)	0.701
Digit vigilance (mean)	456.2 (50.9)	480.9 (56.9)	<0.001	477.2 (55.7)	479.4 (56.3)	0.843
Fluctuating attention
Reaction time (CV) (%)	16.9 (5.1)	16.6 (4.5)	0.598	58.6 (22.0)	53.9 (19.6)	0.258
Choice reaction time (CV) (%)	17.7 (3.6)	18.9 (3.8)	0.008	101.8 (34.1)	100.4 (33.6)	0.831
Digit vigilance (CV) (%)	14.7 (4.1)	16.1 (3.7)	0.004	76.9 (22.9)	77.1 (21.6)	0.975
Executive function
One touch stocking (problems solved)	15.9 (3.1)	14.0 (4.3)	<0.001	14.6 (3.4)	14.2 (4.1)	0.594
Semantic fluency (number of animals in 90 s)	24.2 (6.0)	21.5 (6.5)	0.002	23.2 (7.2)	20.8 (5.1)	0.046
Hayling score	5.9 (1.5)	5.2 (1.6)	0.001	5.3 (1.6)	5.2 (1.7)	0.796
Brixton score	5.2 (1.9)	4.5 (2.3)	0.020	4.4 (2.3)	4.6 (2.4)	0.749
Memory
Pattern recognition memory (number correct)	20.8 (2.3)	19.9 (2.7)	0.013	20.2 (2.7)	19.6 (2.6)	0.272
Spatial recognition memory (number correct)	16.2 (1.9)	15.4 (2.1)	0.002	15.8 (2.1)	15.1 (2.0)	0.107
Paired associate learning (mean trials to success)	1.8 (0.54)	2.1 (0.84)	0.006	2.0 (0.72)	2.1 (0.91)	0.997
Visuospatial
Pentagon copying	1.9 (0.2)	1.8 (0.34)	0.067	1.8 (0.34)	1.9 (0.22)	0.078
MoCA item 1	4.4 (0.65)	4.0 (1.1)	0.005	4.1 (1.2)	4.2(1.1)	0.678

### Associations between gait and cognition

#### Effect of pathology

**Figure 1:**
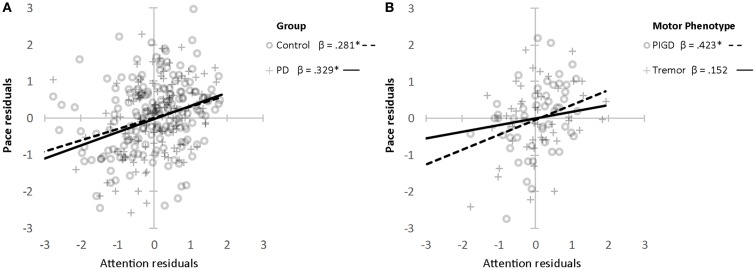
**Association between attention and pace domain of gait (*z* scores) for controls and PD (A) and PIGD and TD (B)**. *Denotes significance at *P* < 0.001. Age, sex, and NART entered as covariates.

Associations between gait and cognition for controls, PD, and PIGD groups are shown in Table 4 (partial correlations), Table 5 (multivariate regression analysis), and Figure 1 (residual plots adjusted for age, sex, and NART for attention and pace for all participants, and associated β-values). Partial correlations identified cognitive correlates for four gait domains in PD (including PIGD) and two gait domains in controls. Multivariate linear regression analyses for PD and controls show an association between faster *pace* and higher scores for *attention*, with the same amount of total variance explained in both groups (29.6%) (see also comparable regression slopes for residuals in Figure 1). For PD, there was also a significant association between more effective *postural control* with better *working memory* explaining 16.3% of total variance, and increased *gait variability* was significantly associated with lower scores for *global cognition*, measured by the MoCA. For controls, *postural control* was also associated with better *attention* although only 6.5% of total variance in the model was explained. Relative to controls, higher β-values for PD for all associations were expressed.

**Table 4:** **Partial correlations for cognitive and gait domains for all participants**.

	Controls (*n* = 184)	PD (*n* = 121)	PIGD (*n* = 55)
Pace	0.31 (*P* < 0.001) A	0.36 (*P* < 0.001) A	0.43 (*P* = 0.003) A
	0.23 (*P* < 0.001) EF	0.20 (*P* = 0.038) EF	
	0.23 (*P* = 0.002) VM	0.28 (*P* = 0.004) WM	
		0.21 (*P* = 0.027) VS	
Rhythm			−0.37 (*P* = 0.010) EF
Asymmetry			
Variability		0.21 (*P* = 0.024) GC	
Postural control	0.17 (*P* = 0.018) A	0.30 (*P* = 0.001) WM	0.32 (*P* = 0.029) WM

*Significant associations only: age, sex, and NART included as covariates*.

*EF, executive function; A, attention; WM, working memory; VS, visuospatial; GC, global cognition*.

**Table 5:** **Summary of regression analyses for significant cognitive variables and gait domains for all participants**.

	Gait domain	Cognitive domain	β	*P*	Adjusted *R*^2^	ANOVA	*P*
Control	Pace^a^	Attention	0.281	<0.001	0.296	*F*(5, 173) = 15.4	<0.001
		Visual memory	0.205	0.003			
	Postural control^a^	Attention	0.183	0.018	0.065	*F*(4, 174) = 4.1	0.003
PD (*n* = 105^a^, *n* = 115^b^)	Pace^a^	Attention	0.329	<0.001	0.296	*F*(5, 100) = 9.8	<0.001
		Working memory	0.256	0.007			
	Postural control^a^	Working memory	0.332	0.001	0.163	*F*(4, 101) = 6.1	<0.001
	Variability^b^	Global cognition (MoCA)	0.289	0.024	0.074	*F*(4, 107) = 3.2	0.016
PIGD (*n* = 55)	Pace^a^	Attention	0.423	0.003	0.196	*F*(4, 44) = 3.9	0.008
	Rhythm^a^	Executive function	−0.468	0.010	0.141	*F*(4, 44) = 2.7	0.041
	Postural control^a^	Working memory	0.321	0.029	0.236	*F*(4, 44) = 4.7	0.003

*^a^For Model, gait domains were regressed on five cognitive domains: attention and fluctuating attention, visual memory, executive function, visuospatial function, and working memory*.

*^b^For Model, MoCA was the only cognitive variable entered. For all models age, sex, and years of education were entered in the first block*.

#### Effect of motor phenotype

Associations were evident for the PIGD but not the TD phenotype. Partial correlations show a strong and positive relationship between *pace* and *attention*, *postural control* and *working memory*, and *rhythm* was negatively correlated with *executive function* showing that participants who walked with a faster cadence had poorer executive function. These relationships held in the regression analysis where β-values for PIGD associations were stronger than for PD or controls. Figure 1 shows a clear effect of motor phenotype, whereby the positive regression slope is steeper for PIGD compared to TD.

## Discussion

To our knowledge this is the first study to comprehensively explore the relationship between independent features of gait and cognition in a large cohort of early PD. Our findings support a significant association between cognition and gait with early stage PD (*n* = 121), which differs with respect to healthy, age-matched controls. The associations were dominated by the PIGD motor phenotype. Importantly, gait impairments were sensitive to motor phenotype discriminating between groups in contrast to cognitive impairments suggesting that shared substrates have a different temporal course. Longitudinal studies will verify the role of gait as a surrogate biomarker of cognitive impairment in PD.

### Early PD pathology and motor phenotype: Impact on gait and cognition

Gait was significantly different in PD compared to controls for 13 of the 16 gait characteristics we tested. Step velocity variability, swing time, and step width were not significantly different, suggesting that these features are preserved at this early stage. Findings from our study partly concur with the only other detailed examination of gait in early PD that we are aware of. Thirty five participants with *de novo* PD (who were not defined by phenotype) showed a significantly slower and more variable gait compared with controls, along with a decrease in stride length and increase in stride time, asymmetry, and double support time (Baltadjieva et al., 2006).

Compared with the TD group, participants with PIGD phenotype were significantly slower and presented with a shorter step length and increased variability of step timing and stance timing. Between-group differences in gait variability were not as strong as anticipated, given its sensitivity to early (Mirelman et al., 2011) and established pathology (Hausdorff et al., 2003), which may be partly due to testing protocol. We report findings from single-task gait, and an increase in gait variability is most often detected during stress testing, such as dual task or fast walking conditions (Amboni et al., 2012). Cognitive outcomes for PD were overall significantly worse than for controls, concurring with earlier work (Muslimovic et al., 2005; Domellof et al., 2011) and in agreement with those reported for the larger ICICLE cohort (Yarnall et al., 2014). However, unlike motor impairment, cognitive deficit was not significantly different between phenotypes in this early cohort, as reported elsewhere (Muslimovic et al., 2005). Interestingly, semantic fluency was more impaired in the PIGD group compared to the TD participants; this test has been shown to predict future dementia in early PD (Williams-Gray et al., 2007).

### Relationship between gait and cognition in PD and controls: Evidence for common and distinct features

For control, PD, and PIGD participants the strongest and most consistent associations were found for the pace domain of gait and attention, which is underpinned by frontal and prefrontal activity that are mediated by dopaminergic as well as cholinergic substrates (Rochester et al., 2012). There may be different reasons for this. Firstly, gait is a goal directed activity realized through activation of attentional circuits, which project from dorsolateral prefrontal cortex to the dorsolateral head of the caudate nucleus, with dopamine and acetycholine involved in signaling at various levels of the circuit (Calabresi et al., 2006). Secondly, changes to motor control occur early in PD as movement degrades from being responsive and automatic to slow, and voluntary, thus, requiring compensatory cognitive (particularly attentional) effort (Redgrave et al., 2010). We were surprised that the pace–attention relationship was not evident for the TD phenotype given that it was significant for controls. The reasons for this are unclear. We controlled for age and sex in analysis, ruling out these as potential confounders. The control group (*n* = 184) may have presented with higher levels of vascular burden, which may subtly impact on the relationship, however, this is speculative. Inspection of the slopes suggests a similar trend for TD and controls, and we may have been underpowered to detect a significant difference. Cognition also played a consistent role in postural control for all participants; however, this was underpinned by attention in controls and working memory in PD and PIGD phenotype. Attention made only a small contribution in controls (6%) compared to working memory in PD (16%), which increased in PIGD to 24%. Cognition, particularly attention and executive function, is known to contribute to postural control in older adults with some evidence in PD (Schoneburg et al., 2013). The role of working memory is interesting. Working memory in this study was measured using the digit span (forward), which also reflects short-term memory. Given that its contribution to PD was dominated by the PIGD phenotype this may reflect early changes in features consistent with amnestic function although longitudinal studies are required to test this hypothesis. Alternatively, it may reflect amyloid deposition, given studies showing lower CSF amyloid in PIGD (Alves et al., 2013) and negative correlation between memory scores and Aβ (Alves et al., 2010).

Variability was associated with global cognition as reported in older adults (Lord et al., 2013a; Verlinden et al., 2014) but surprisingly not related to a specific cognitive domain. This is possibly due to the early disease presentation, and the association may become more specific as disease advances.

### PIGD phenotype dominates the relationship between gait and cognition

Associations between gait and cognition were clearly different for phenotype despite comparable cognitive outcomes, which allows us to conjecture about the relationships we observed. If associations were due to co-incident gait/cognitive pathology, they would be evident across the continuum of disease (albeit weaker for TD), and not restricted to PIGD. An alternate explanation is that attention and executive function were used to maintain gait performance in people with PIGD, but not required to the same extent in TD because gait is less impaired. Associations may also point to a shared gait/cognition substrate for PIGD phenotype but not for TD; however, longitudinal data are required to confirm this.

We were surprised to find that rhythm was inversely associated with cognition (executive function) for PIGD given that it is primarily considered a subcortical feature of gait mediated by spinal and brainstem mechanisms and that values for rhythm characteristics (step time, stance time, and swing time) were not sensitive to motor phenotype in this early cohort. However, the rhythm domain of gait has been shown to be sensitive to memory decline in older adults (Verghese et al., 2007) and also to information processing (Verlinden et al., 2014) suggesting that this is not an aberrant finding.

Results from this study concur with our earlier work in more advanced cohorts, which indicate a preferential contribution of attention and executive function to gait, and a dominant effect of dopaminergic medication on the relationship between cognition and gait (Rochester et al., 2008; Lord et al., 2010). The interaction between gait and cognition is complex because both evolve and are likely to be associated differently over the course of the disease. Gait domains that are strongly correlated with cognition at baseline may not be predictive of future cognitive and motor decline. Williams-Gray et al. (2007) identified the prognostic strength of posterior cortical origin deficits (semantic fluency, intersecting pentagons) for predicting dementia in PD, although frontostriatal deficits were more striking at baseline. Results from this study suggest that gait is more sensitive than cognition to PD phenotype; however, longitudinal follow up of ICICLE-GAIT data will map the evolution of gait and cognitive dysfunction as disease progresses and identify the putative role of gait as a surrogate biomarker for cognitive decline.

### Study strengths and limitations

A key strength of this hypothesis-driven prospective study is the recruitment of a well described community-representative incident cohort and an age-matched control group. Limitations include the small sample size for phenotype analysis and the fact that not all PD participants were treatment-naïve, and the beneficial effects of dopaminergic replacement therapy may have biased results. We did not use a dual task testing paradigm because preliminary analysis (not reported) found the dual task results to be highly comparable to single task. This may be due in part to our dual task testing protocol, which controls for baseline cognitive capacity (Rochester et al., 2014), and as a result sensitivity may be lower than previous reports, which have detected very early signals for gait variability (Mirelman et al., 2011). The cognitive domains we used in this study were not completely independent of each other; for example, the correlation between attention and PRM and SRM is highly significant, and this interdependence has to be kept in mind when interpreting the results. We carried out a preliminary principle component analysis for our cognitive variables but were unable to identify discrete factors without cross-loadings, and so rather than taking a data-driven approach we mapped each cognitive outcome onto well-established domains. This also ensured consilience with recently published work (Yarnall et al., 2014). We did not include a language domain in our analysis. The tests we used for language comprise single items from the MoCA and MMSE and we did not find any convincing correlations with gait in preliminary analysis. We did not report lastly, we did not control for depression (GDS) in analysis that compared PD and controls, although we have previously argued for its association with gait possibly mediated via cognition (Lord et al., 2013b). Analysis of our longitudinal data set will test this hypothesis further.

## Conclusion

Cognitive function is associated with gait in early PD, and this association is driven by the PIGD motor phenotype. The association is strongest for attentional control and pace and also includes other cognitive and gait domains. Future analysis on this longitudinal cohort will enhance understanding of the complex interaction between gait and cognition in PD.

## Conflict of Interest Statement

The authors declare that the research was conducted in the absence of any commercial or financial relationships that could be construed as a potential conflict of interest.
